# Editorial Board

**DOI:** 10.1177/2055116917690954

**Published:** 2017-03-14


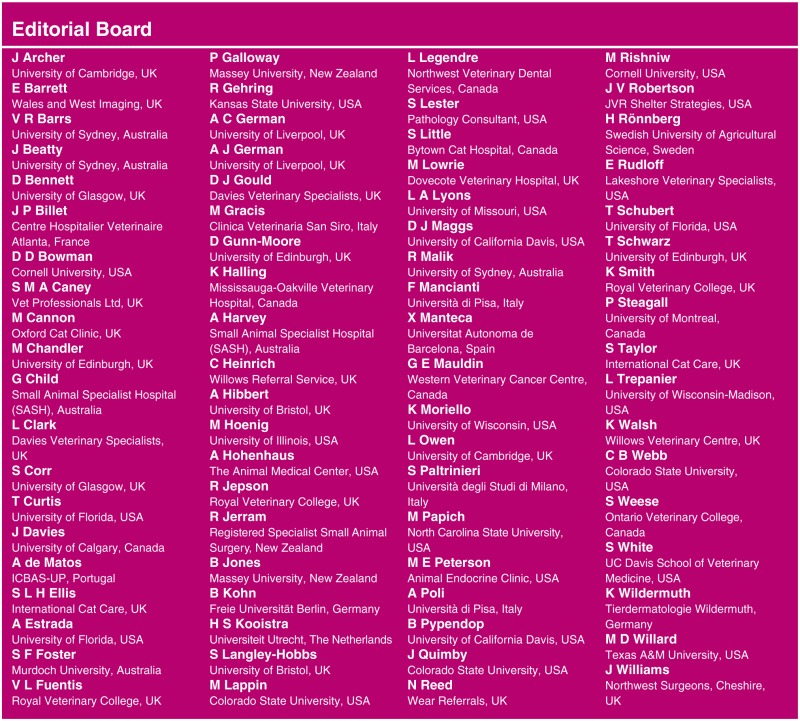


**Editors**

**A H Sparkes**

International Society of Feline Medicine, Veterinary Division of International Cat Care, High Street, Tisbury, Wiltshire SP3 6LD, UK

Email: andy@icatcare.org

**M Scherk**

catsINK, Vancouver, BC, Canada V5N 4Z4

Email: hypurr@sagepub.co.uk hypurr@aol.com

**Editorial Team**

**C Bessant** (Executive Editor)

**M Melling** (Managing Editor)

**A Tansley** (Assistant Editor)

International Society of Feline Medicine, Veterinary Division of International Cat Care, High Street, Tisbury, Wiltshire SP3 6LD, UK

Email: jfms@icatcare.org; jfmsclinicalpractice@icatcare.org

